# Elevated lymphoma risk in patients with chronic pruritus and chronic prurigo

**DOI:** 10.1111/ddg.70057

**Published:** 2026-04-06

**Authors:** Svenja Royeck, Henning Olbrich, Steffen Koschmieder, Claudia Zeidler, Felix Witte, Konstantin Agelopoulos, Henning Wiegmann, Michael Storck, Johannes Benedict Oehm, Ralf J. Ludwig, Sonja Ständer

**Affiliations:** ^1^ Section Pruritus Medicine Department of Dermatology and Center for Chronic Pruritus (KCP) University Hospital Münster Münster Germany; ^2^ Department of Dermatology University‐Hospital Schleswig‐Holstein (UKSH) Lübeck Germany; ^3^ Department of Haematology Oncology Hemostaseology and Stem Cell Transplantation Faculty of Medicine RWTH Aachen University Aachen Germany; ^4^ Center for Integrated Oncology Aachen Bonn Cologne Düsseldorf (CIO ABCD) Aachen Germany; ^5^ Institute of Medical Informatics University of Münster Münster Germany; ^6^ Lübeck Institute of Experimental Dermatology University of Lübeck Lübeck Germany; ^7^ Institute and Comprehensive Center for Inflammation Medicine University of Lübeck Lübeck Germany

**Keywords:** chronic prurigo, chronic pruritus, cohort study, lymphoma, real‐world data, TriNetX

## Abstract

**Background and objectives:**

Chronic pruritus (CP) and chronic prurigo (CPG, including prurigo nodularis) are prevalent, debilitating diseases with diverse comorbidities, including malignancies. This retrospective cohort study evaluated the prevalence and risk of lymphoma in CP and CPG patients, including key risk factors, and sex‐ and ancestry‐related disparities.

**Patients and methods:**

We utilized electronic health records from the TriNetX US Collaborative Network (2011–2024), including 1,032,281 CP and 124,611 CPG patients (without prior lymphoma), matched to non‐CP/non‐CPG controls. Lymphoma risk was evaluated up to 5 years post‐diagnosis. Sensitivity, sex‐ and ancestry‐stratified analyses were performed. Multivariate analyses identified risk factors.

**Results:**

Lymphoma developed in 0.29 % of CP (controls: 0.10 %, HR 2.90, 95 % 95 % CI: 2.71–3.12) and 0.30 % of CPG patients (controls: 0.10 %, HR 2.85, 95 % CI: 2.33‐3.48; both p < 0.0001). The HR for cutaneous lymphoma was 6.62 (95 % CI 5.02–8.72) for CP and 10.95 (95 % CI 4.39–27.35) for CPG (p < 0.0001 each). Findings remained robust across all sensitivity analyses. Significant lymphoma risk factors included elevated serum lactate dehydrogenase, beta 2‐microglobulin, male sex, immunosuppressant use, and White ancestry.

**Conclusions:**

CP and CPG patients have an increased lymphoma risk, linked to specific biomarkers, highlighting the importance of increased clinical vigilance in their management.

## INTRODUCTION

Chronic pruritus (CP; ≥ 6 weeks), with a prevalence of 16.8 % in the working population, is a burdensome symptom which significantly reduces the quality of life (QoL) of affected individuals and places a considerable economic burden on healthcare systems.^1^, ^2^

Chronic pruritus constitutes one of the three cardinal clinical features of chronic prurigo (CPG; estimated prevalence 0.1 %), a disorder characterized by chronic pruritus in combination with multiple pruriginous lesions and persistent scratching behavior.^3^, ^4^ CP and CPG, including its most common subtype prurigo nodularis (PN), affect individuals of all age groups but are more prevalent among older adults and frequently occur in association with a broad spectrum of comorbidities.^5^, ^6^, ^7^, ^8^ In fact, some studies showed a higher prevalence of comorbidities including neoplasms.^6^, ^8^, ^9^

Moreover, CP and CPG may precede the clinical manifestation of malignancies such as solid tumors and hematolymphoid neoplasms^10^, ^11^, ^12^, ^13^, ^14^ and cutaneous T‐cell lymphoma (CTLC)^15^, ^16^ by months to years, potentially serving as an early clinical indicator or premonitory symptom.^17^, ^18^ A nationwide Danish cohort study investigating 12,813 pruritus patients identified a two‐fold increased cancer incidence during the initial three‐month post‐diagnosis period, with a cumulative 1‐year incidence of 1.63 %. Incidence rates declined exponentially thereafter and approximated those of the general population after 10 years of follow‐up.^18^ In this study, pruritus demonstrated a significant association with hematologic malignancies, with the highest standardized incidence ratio observed for Hodgkin lymphoma (HL).^18^ The prevalence of CP in lymphoma ranges from more than 15 % in non‐Hodgkin lymphoma (NHL) up to 30 % in HL.^19^, ^20^

While numerous case reports and case series have described associations between CP, CTCL and systemic lymphoma,^21^, ^22^, ^23^, ^24^, ^25^, ^26^, ^27^, ^28^, ^29^, ^30^, ^31^, ^32^, ^33^ the link between CPG and lymphoma has been reported only in a limited number of cases.^34^, ^35^, ^36^, ^37^ Consequently, the relationship between CPG and lymphomagenesis remains poorly understood. Comprehensive long‐term data on the risk of lymphoma in patients with chronic pruritus (CP) and chronic prurigo (CPG), particularly studies incorporating relevant risk factors, remain limited. In particular, there is a lack of studies that adjust for potential confounders using detailed matching procedures, evaluate sex‐ and ancestry‐related disparities, and examine the temporal relationship between the diagnosis of CP or CPG and subsequent lymphoma onset. To address these knowledge gaps, a large‐scale longitudinal study was conducted using electronic health records (EHRs) from the federated TriNetX database. The study analyzed 1,032,281 EHRs from patients with CP, 124,611 EHRs from patients with CPG including PN, and an equal number of matched non‐CP and non‐CPG controls.

## PATIENTS AND METHODS

### Study design and database

This propensity‐score‐matched retrospective cohort study utilized the TriNetX US Collaborative Network, which provides extensive EHRs and detailed covariate data.^38^ EHRs from patients with CP, CPG including PN, and matched controls were analyzed to assess lymphoma risk. The analysis differentiated between CTCL and systemic lymphomas using ICD‐10‐CM codes, applying propensity‐score matching (PSM) for comparability. Both lymphoma groups were considered in overall risk assessment. Three sensitivity analyses and subgroup analyses by sex and ancestry were performed to test robustness and identify disparities. Cox proportional hazards (CPH) analysis assessed risk factors using combined CP and CPG cohorts. The study design is depicted in Figure 1. The study followed STROBE guidelines.^39^

**FIGURE 1:**
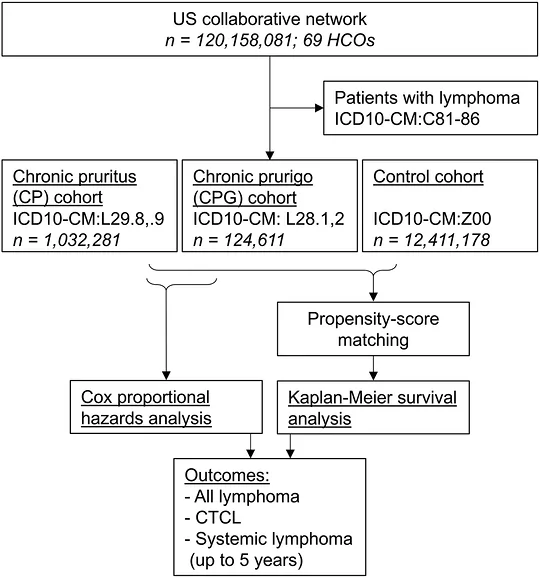
Study outline. After identification of n = 120,158,081 electronic health records from 69 healthcare organizations (HCOs) within the US collaborative network of TriNetX and exclusion of patients with lymphoma (defined by ICD‐10‐CM codes C81–C86), patients with chronic pruritus (CP; defined by ICD codes L29.8 and L29.9), chronic prurigo (CPG; defined by ICD codes L28.1 and L28.2), and a control cohort were selected. Propensity score matching was performed to assess the 5‐year risk of lymphoma development (overall lymphoma, systemic lymphoma, and cutaneous T‐cell lymphoma) using the Kaplan–Meier method. Cox proportional hazards analysis was applied to identify relevant risk factors for lymphoma development in patients with CP and CPG, including laboratory biomarkers.

### Study population

The TriNetX database included over 120 million patient EHRs from 69 US healthcare organizations (HCOs). Adults (≥ 18 years) with CP (ICD‐10‐CM: L29.8, L29.9) or CPG (ICD‐10‐CM: L28.1, L28.2) were included, excluding those with overlapping codes. Controls were selected from patients with general examination encounters (ICD‐10‐CM: Z00) and without CP/CPG codes. The index event was the diagnosis of CP/CPG or the control encounter. Outcomes prior to the index event, including lymphoma, were excluded.

### Covariates

Propensity score matching was applied to reduce confounding by matching patients on age, sex, White ancestry, overweight/obesity, and nicotine dependence. Sensitivity analysis S3 included additional variables: infectious diseases, autoimmune diseases, and immunosuppressant use. Matching was 1:1. Baseline characteristics before and after matching are reported in Table 1. Additional predictors, including laboratory values (LDH, β2‐microglobulin, hemoglobin, CRP, albumin), were analyzed using CPH in the combined CP and CPG cohorts.

**TABLE 1:** Baseline characteristics before and after propensity score matching in chronic pruritus (CP) and chronic prurigo (CPG) (cases) and their respective controls. Cohort assembly was performed with strict exclusion of individuals with a pre‐existing diagnosis of lymphoma at the time of inclusion. Propensity score matching was conducted using a covariate matrix that included age at index (continuous), female sex (binary), self‐reported White ancestry (binary), and lifestyle‐related variables (overweight and obesity, nicotine dependence, and personal history of nicotine dependence).

Characteristic Name	ICD‐10	CP		CPG		Control	
Total n		1,032,281		124,611		12,411,178	
Age at Index (years)		50.70	18.17	53.09	16.40	46.27	17.91
		*n*	*%*	*n*	*%*	*n*	*%*
Ancestry (White)		690,839	66.92%	78,999	63.40%	7,693,060	61.99%
Female		597,397	57.87%	70,400	56.50%	6,610,172	53.26%
Male		308,273	29.86%	49,501	39.72%	5,133,379	41.36%
Ancestry (Black)		170,104	16.48%	14,528	11.66%	1,585,952	12.78%
Ancestry (Hispanic)		116,432	11.28%	8,640	6.93%	966,961	7.79%
Overweight and obesity	E66	214,218	20.75%	24,896	19.98%	1,199,551	9.67%
Nicotine dependence	F17	134,578	13.04%	14,644	11.75%	701,352	5.65%
Personal history of nicotine dependence	Z87.891	102,484	9.93%	9,910	7.95%	535,135	4.31%

### Outcomes

Outcomes were defined by ICD‐10‐CM codes: All lymphomas (C81–C86), CTCL (C84.0, C84.1, C84.A, C86.6), and systemic lymphomas (C81–C85, excluding CTCL codes). Outcomes were pre‐specified.

### Primary, sensitivity and subgroup analyses

The primary analysis included outcomes from one day to 5 years post‐index. Sensitivity analysis S1 considered only outcomes occurring 3 months to 5 years post‐index to account for potentially undiagnosed or uncoded diseases at baseline. A supplemental analysis extended follow‐up to 10 years. S2 included only patients with at least one encounter in the year prior to index to enhance PSM validity. S3 used more extensive PSM but limited to recent cases due to computational constraints. Sex‐ and ancestry‐stratified analyses applied primary definitions but stratified by self‐reported sex (female or male) or by self‐reported ancestry (White, Black/African American or Hispanic/Latino).

### Statistical analysis

Propensity scores were estimated for each patient using logistic regression, with exposure status specified as the dependent variable. The analysis was performed in Python using the scikit‐learn library. One‐to‐one matching was performed using a greedy nearest‐neighbor algorithm, applying a caliper width of 0.1 pooled standard deviations of the logit‐transformed propensity score. Following matching, baseline characteristics were reassessed and reported. Differences were evaluated using t‐tests for continuous variables and z‐tests for binary or categorical variables. Relative risks and risk differences were calculated. Survival analysis was performed using the Kaplan–Meier method in R's Survival package (R Foundation for Statistical Computing, Vienna, Austria, version 3.2‐3) and validated against outputs from SAS version 9.4 (SAS, Cary, NC). The proportionality assumption was evaluated using the cox.zph() function within R's Survival package. Log‐rank tests were used to compare Kaplan–Meier curves. Univariate Cox proportional hazards regression was employed to estimate hazard ratios with 95 % confidence intervals (95 % CI). To mitigate bias from multiple testing, the Bonferroni correction was applied, adjusting the significance level to α = 0.0167. Additionally, risk factors for development of lymphoma were analyzed using CPH analysis considering all predictors used in the extended PSM in analysis S3, as well as clinically relevant laboratory values. Cox regression coefficients were exponentiated to express hazard ratios with estimated 95 % CIs and differences between groups were compared with Wald tests. Participants were censored after their latest available record.

### Ethics statement

The data reviewed is a secondary analysis of existing data, does not involve intervention or interaction with human subjects, and is de‐identified per the de‐identification standard defined in Section §164.514(a) of the HIPAA Privacy Rule. TriNetX is a federated network which facilitates collaboration between industry including pharmaceutical companies and research organizations, academia, health care organizations (HCOs). TriNetX operates under a security and governance framework that provides a federated platform for real‐world data (RWD), including electronic health records (EHRs), and is compliant with the Health Insurance Portability and Accountability Act (HIPAA), the General Data Protection Regulation (GDPR), and the Lei Geral de Proteção de Dados (LGPD). Accordingly, Institutional Review Board approval was not required for this study.

## RESULTS

### Baseline characteristics

In the primary analysis, three cohorts were identified: patients with CP (n = 1,032,281), patients with CPG (n = 124,611), and controls without CP or CPG (n = 12,411,178). The mean age was 50.7 ± 18.2 years in the CP cohort, 53.1 ± 16.4 years in the CPG cohort, and 46.3 ± 17.9 years among controls (standardized difference [std. diff.] = 0.246 for CP and 0.397 for CPG vs controls). Females accounted for 66.9 % of CP, 56.5 % of CPG, and 53.3 % of controls (std. diff. = 0.282 for CP and 0.065 for CPG vs controls). Both CP and CPG cohorts exhibited higher prevalences of lifestyle‐related risk factors, including overweight or obesity (20.8 % in CP, 20.0 % in CPG, and 9.7 % in controls; std. diff. = 0.312 for CP and 0.293 for CPG vs controls). After propensity score matching, baseline characteristics were well balanced, with comparable mean age, sex distribution, and prevalences of overweight or obesity and nicotine dependence across groups (all std. diff. < 0.001). Detailed cohort characteristics are presented in Table 1.

### Increased risk of systemic and overall lymphoma development following chronic pruritus and chronic prurigo diagnoses, particularly in males, patients taking immunosuppressants, and those of White ancestry

Incident systemic lymphomas following a diagnosis of CP or CPG were assessed over a 5‐year follow‐up period. In the primary analysis, 0.26 % of patients with CP developed systemic lymphoma (including HLs and systemic NHLs) compared with 0.10 % of controls. This difference corresponded to a hazard ratio (HR) of 2.74 in the Kaplan–Meier analysis (95 % CI 2.55–2.95; p < 0.0001) (Figure 2; online supplementary Figure S1), indicating that patients with CP had an approximately 3‐fold increased risk of developing systemic lymphoma. This finding was corroborated in the analysis of all lymphomas combined, which yielded a hazard ratio of 2.90 (95 % CI 2.55–2.95; p < 0.0001). In the CPG cohort, an increased risk of systemic lymphoma was likewise observed, with an absolute risk of 0.26 % in the primary analysis. In the Kaplan–Meier analysis, this corresponded to a hazard ratio (HR) of 2.60 (95 % CI 2.12–3.20; p < 0.0001) (Figure 3; online supplementary Figure S2). Similar results were obtained in the combined analysis of all lymphomas (HR 2.85; 95 % CI 2.33–3.48; p < 0.0001). These findings were consistent across all sensitivity analyses (online supplementary Figures S3, S4).

In a multivariable Cox proportional hazards analysis of the combined CP and CPG cohort, male sex (HR 1.65; 95 % CI 1.53–1.78; p < 0.0001; Figure 4), treatment with immunosuppressive medications (HR 1.31; 95 % CI 1.14–1.51; p < 0.0001), and White ancestry (HR 1.27; 95 % CI 1.17–1.38; p < 0.0001) were identified as independent predictors of systemic lymphoma development. Comparable results were observed in the analysis of all lymphomas combined. These findings were confirmed in sex‐ and ancestry‐stratified analyses (online supplementary Tables S1, S2).

### Increased cutaneous lymphoma risk in patients with chronic pruritus and chronic prurigo

In separate analyses of cutaneous lymphoma (including mycosis fungoides, Sézary syndrome, and primary cutaneous CD30‐positive lymphoproliferative disorders), distinct from systemic lymphoma, increased risks were observed for both CP and CPG cohorts. The absolute risk was 0.037 % in the CP cohort and 0.045 % in the CPG cohort, compared with 0.006 % in controls. This corresponded to HRs of 6.62 (CP; 95 % CI 5.02–8.72; p < 0.0001) (Figure 2) and 10.95 (CPG; 95 % CI 4.39–27.35; p < 0.0001) (Figure 3). This was consistent in the sensitivity analyses. In the Cox proportional hazards analysis, treatment with immunosuppressive agents (HR 3.01; 95 % CI 2.19–4.12; p < 0.0001) (Figure 4) and male sex (HR 2.06; 95 % CI 1.69–2.51; p < 0.0001) were identified as strong predictors of cutaneous lymphoma. In contrast to systemic lymphoma, White ancestry was associated with a reduced risk (HR 0.72; 95 % CI 0.59–0.88; p < 0.0001).

**FIGURE 2:**
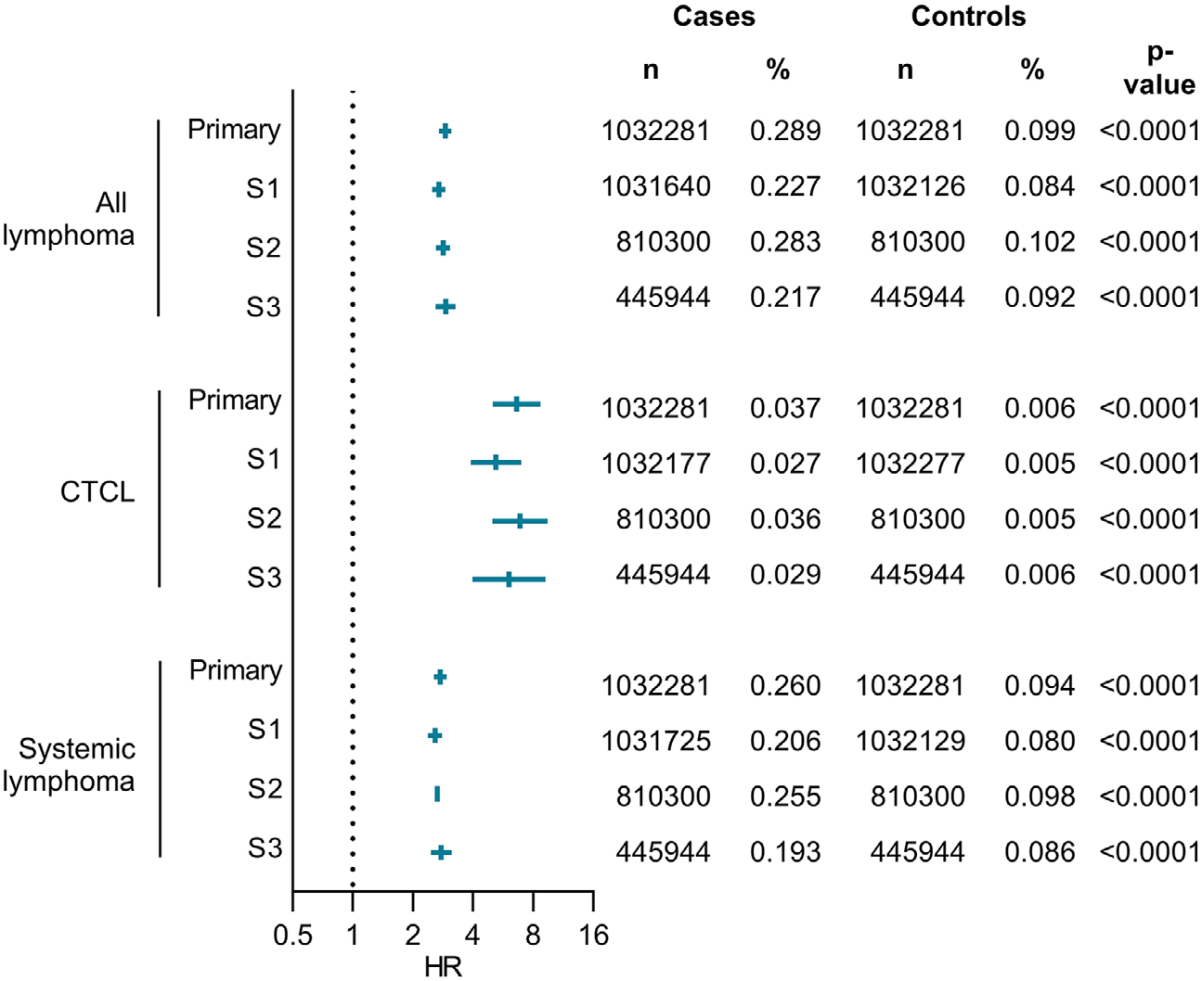
Risk of lymphoma development in patients with chronic pruritus compared with controls. The figure displays the hazard ratios (HRs) for lymphoma development (overall lymphoma, cutaneous T‐cell lymphoma [CTCL], and systemic lymphoma) following the diagnosis of chronic pruritus (cases) compared with controls (patients without chronic pruritus), as assessed in the primary analysis and in all three sensitivity analyses (S1–S3) using the Kaplan–Meier method.

**FIGURE 3:**
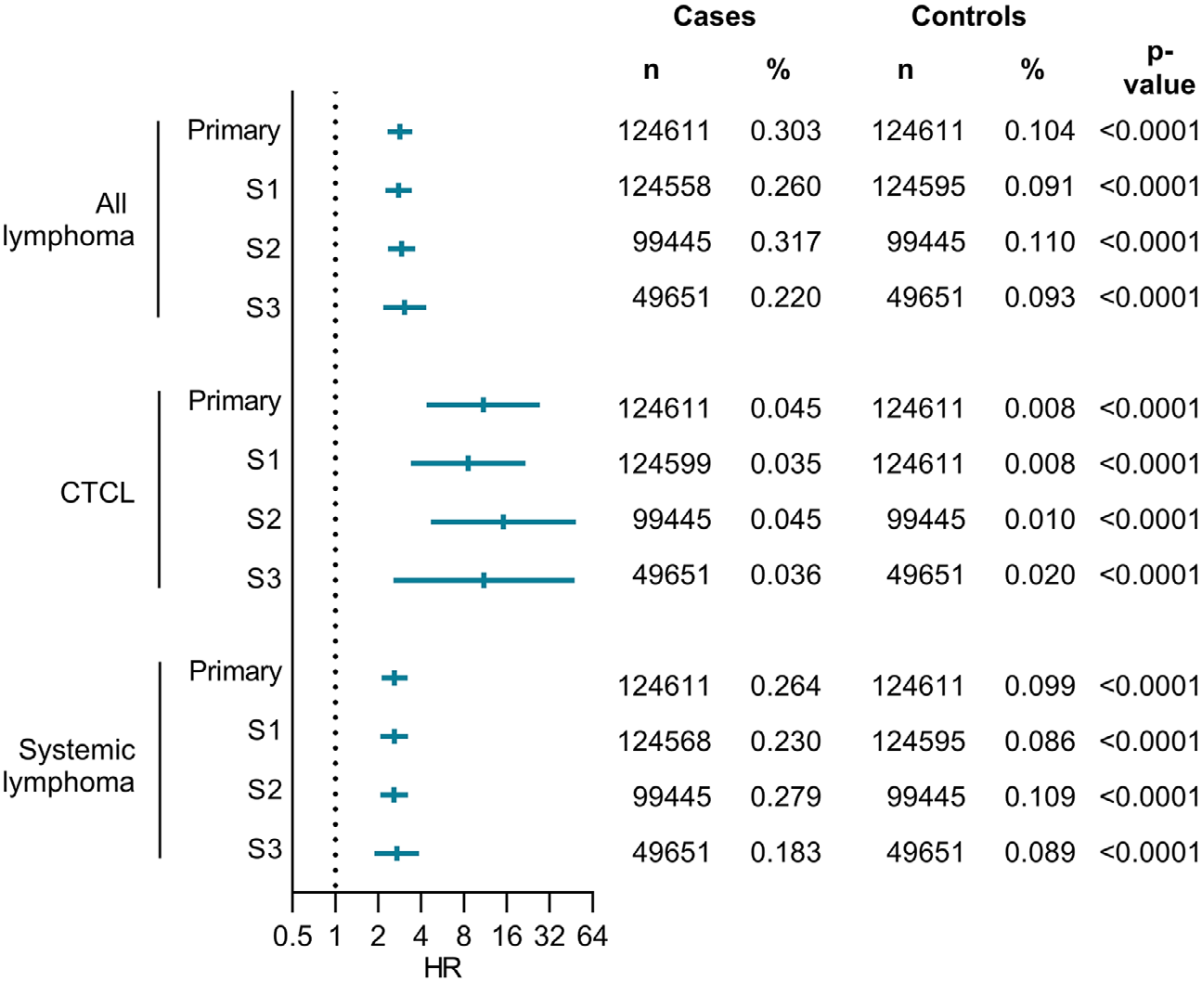
Risk of lymphoma development in patients with chronic prurigo compared with controls. The figure displays the hazard ratios (HRs) for lymphoma development (overall lymphoma, cutaneous T‐cell lymphoma [CTCL], and systemic lymphoma) following the diagnosis of chronic prurigo (cases) compared with controls (patients without chronic prurigo), as assessed in the primary analysis and in all three sensitivity analyses (S1–S3) using the Kaplan–Meier method.

**FIGURE 4:**
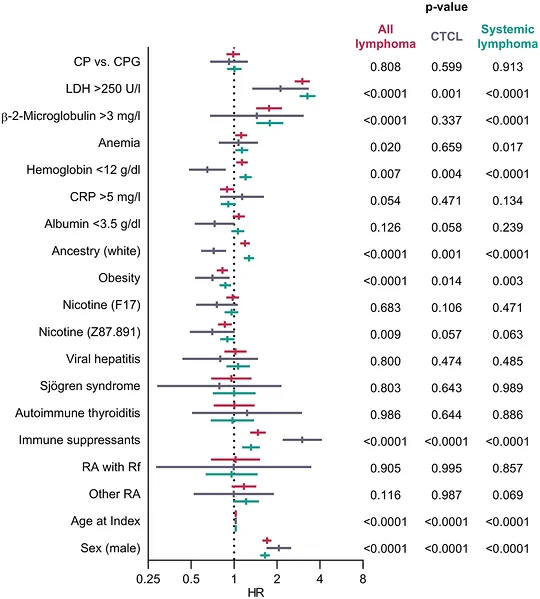
Risk factors for lymphoma development in patients with chronic pruritus and chronic prurigo. The figure presents the hazard ratios (HRs) of risk factors associated with lymphoma development (overall lymphoma, cutaneous T‐cell lymphoma [CTCL], and systemic lymphoma) based on Cox proportional hazards analysis using the combined cohorts of patients with chronic pruritus (CP) and chronic prurigo (CPG). All variables included in the propensity score matching were considered, in addition to elevated serum lactate dehydrogenase (LDH; > 250 U/L), increased serum β2‐microglobulin (> 3 mg/l), anemia (ICD‐10‐CM: D64), low hemoglobin (<12 g/dl), elevated C‐reactive protein (CRP; > 5 mg/l), and hypoalbuminemia (< 3.5 g/dl).

### Key laboratory parameters linked to an elevated lymphoma risk in patients with chronic pruritus and chronic prurigo

Elevated serum LDH (> 250 U/L) was the strongest laboratory predictor of lymphoma development in patients with CP and CPG. It was associated with an approximately 3‐fold increased risk of all lymphomas (HR 3.01; 95 % CI 2.66–3.40; p < 0.001) and systemic lymphoma specifically (HR 3.27; 95 % CI 2.88–3.72; p < 0.001). Elevated serum β2‐microglobulin represented the second most relevant laboratory risk factor for all lymphomas (HR 1.76; 95 % CI 1.43–2.17; p < 0.001) and systemic lymphoma (HR 1.78; 95 % CI 1.43–2.21; p < 0.001), but was not associated with cutaneous lymphoma. Low hemoglobin levels (< 12 g/dL), indicative of anemia, were the third most important predictor for all lymphomas (HR 1.14; 95 % CI 1.04–1.25; p < 0.01) and systemic lymphoma (HR 1.20; 95 % CI 1.09–1.33; p < 0.001), but were not significantly associated with cutaneous lymphoma. Additional risk factors are presented separately (Figure 4).

## DISCUSSION

This study demonstrates an increased overall risk of lymphoma development – particularly systemic lymphoma – following the initial diagnosis of CP and CPG, including PN, compared with individuals without CP or CPG. Most interestingly, while clinically different, patients with CP and CPG have a comparable risk of developing lymphoma within 5 years following the initial diagnosis of CP and CPG. Associated risk factors for lymphoma development are sex, ancestry, comorbidities, lifestyle factors, medication use; and laboratory results.

The recognition of CP and CPG as potential indicators of lymphoma risk holds significant clinical implications, necessitating heightened diagnostic vigilance and tailored screening protocols.

To ensure cost‐effective patient management, overdiagnosis should be avoided without delaying the diagnosis of underlying and developing comorbidities that could lead to increased mortality and morbidity.^40^ Our data indicate a five‐year lymphoma risk of 0.3 % in patients with CP and CPG. This aligns with results from a recent study, in which the risk of HL in patients with CP was reported at 0.5 % and the risk of NHL at 0.22 % 5 years after diagnosis, both significantly higher compared to healthy controls (HL risk: 0.24 %, NHL risk: 0.13 %; p < 0.001).^41^ This study also emphasizes that the risk of developing HL and NHL is significantly higher in the first year following a CP diagnosis, compared to the subsequent 5 and 10 years.^41^

While these results are not directly comparable to ours due to methodological differences, both findings highlight the importance of regularly monitoring patients with CP and CPG for hematologic malignancies. This finding should be taken into account in clinical practice, with careful history‐taking to assess additional symptoms and to evaluate the individual course and progression of pruritus. During the physical examination, lymph node stations should be palpated regularly, and laboratory and imaging diagnostics should be repeated at appropriate intervals, as recommended in the current guidelines for the diagnosis and treatment of CP, to facilitate the early detection of hematologic malignancies.^42^, ^43^ Particular attention should be paid to patients with CP and CPG who exhibit risk factors for systemic lymphoma as identified in our analysis, including elevated serum LDH levels (>250 U/L), increased β2‐microglobulin concentrations (>3 mg/L), male sex, treatment with immunosuppressive agents, White ancestry, the presence of anemia, and concomitant infectious or autoimmune diseases.

Interestingly, our data indicate that the most significant laboratory parameter associated with lymphoma occurrence in patients with CP and CPG is an elevated serum LDH level. In another global cohort analysis, elevated serum LDH levels were observed in patients with CP; however, among 3,744 pruritus patients with available serum LDH data, these levels were not associated with an increased risk of hematologic cancers, including HL and NHL.^41^ We hypothesize that this discrepancy may be due to the significantly larger sample size analyzed in our study, as well as the inclusion of more comprehensive data in TriNetX compared to 3 years ago. A major strength of our analysis is the evaluation of a broader range of lymphoma risk factors in patients with CP and CPG using Cox regression analyses.

Several limitations should be considered. The retrospective design and reliance on real‐world data introduce the potential for residual confounding and bias due to unmeasured variables, despite the application of comprehensive PSM. Causality between CP/CPG and lymphoma cannot be established, as unexamined comorbidities, genetic factors, environmental exposures, and gene‐environment interactions may influence the association.

A critical unresolved issue is the temporal relationship between CP, CPG, and lymphoma onset. While lymphoma cases on CP and CPG preceding lymphoma diagnosis by months to years^30^, ^32^ are reported in the literature, our cohort analysis cannot conclusively determine whether CP and CPG represent premonitory symptoms (early indicators of latent lymphoma development), paraneoplastic manifestations (CP and CPG secondary to [still] undiagnosed malignancies),^44^, ^45^, ^46^ or developed completely independent of the lymphoma diagnosis in the context of numerous other underlying or comorbid diseases which are common for CP and CPG.^47^, ^48^, ^49^, ^50^

Despite excluding pre‐existing lymphoma diagnoses, the retrospective design limits causal inference due to potential lead‐time bias and ICD‐10 coding inaccuracies inherent to RWD.

Moreover, another intriguing question for future research would be to investigate and compare the risks and risk factors for different systemic and cutaneous lymphomas in patients with CP and CPG, given the markedly distinct courses and prognoses of the individual entities. However, this question could not be addressed in the present study, as the case numbers for each lymphoma subtype were too low to allow separate analyses for the individual lymphoma entities.

Additional limitations include the lack of histological confirmation, independent diagnostic validation, and unmeasured socioeconomic confounders from healthcare access disparities.

However, the strength of this analysis lies in the large, RWD set that confirms previous reports of increased malignancies in CP and CPG cohorts. Confirming these data in registry data with more biological data is essential to confirm a causal relationship. A more comprehensive understanding would refine diagnostic and therapeutic recommendations, optimizing interdisciplinary patient management.

## FUNDING

This work was supported by the Cluster of Excellence “Precision Medicine in Chronic Inflammation” (EXC 2167), the Collaborative Research Center PANTAU (SFB 1526), the Clinician Scientist School Lübeck (CS012022), and the individual grant LU 877/25‐1, all funded by the Deutsche Forschungsgemeinschaft, as well as by the Schleswig‐Holstein Excellence Chair Program of the State of Schleswig‐Holstein. This work was further supported by the Deutsche Forschungsgemeinschaft (Research Group 2690 PRUSEARCH; STA 1159/4‐2 and 5211 SOMACROSS; STA 1159/7‐1).

## CONFLICT OF INTEREST STATEMENT

H.O. and R.J.L. received travel grants from TriNetX. All other authors declare no conflict of interest.

## Supporting information



Supplementary information
