# Supplementary material for: Elevated lymphoma risk in patients with chronic pruritus and chronic prurigo
Source: J Dtsch Dermatol Ges. 2026 Apr 6;24(11):1588–96. doi: 10.1111/ddg.70057 (PMC13749593; doi:10.1111/ddg.70057)
Supplement: Supplementary file 1 [file DDG-24-1588-s001.docx]

**Supplemental material**

**Elevated lymphoma risk in patients with chronic pruritus and chronic prurigo**

Svenja Royeck*^1^, Henning Olbrich*^2^, Steffen Koschmieder^3,4^, Claudia Zeidler^1^, Felix Witte^1^, Konstantin Agelopoulos^1^, Henning Wiegmann^1^, Michael Storck^5^, Johannes Benedict Oehm^5^, Ralf J. Ludwig*^2,6,7^, Sonja Ständer*^1^

^1^ Section Pruritus Medicine, Department of Dermatology and Center for Chronic Pruritus (KCP), University Hospital Münster, Münster, Germany

^2^ Department of Dermatology, University-Hospital Schleswig-Holstein (UKSH), Lübeck, Germany

^3^ Department of Haematology, Oncology, Hemostaseology and Stem Cell Transplantation, Faculty of Medicine, RWTH Aachen University, 52074 Aachen, Germany

^4^ Center for Integrated Oncology Aachen Bonn Cologne Düsseldorf (CIO ABCD), 52074 Aachen, Germany

^5^ Institute of Medical Informatics, University of Münster, Münster

^6^ Lübeck Institute of Experimental Dermatology, University of Lübeck, Lübeck, Germany

^7^ Institute and Comprehensive Center for Inflammation Medicine, University of Lübeck, Lübeck, Germany

*equal contribution

Corresponding author: Svenja Royeck, Section Pruritus Medicine, Department of Dermatology and Center for Chronic Pruritus (KCP), University Hospital Münster, Münster, Germany; email: Svenja.Royeck@ukmuenster.de

**Table S1**. Sex- and ancestry-stratified risk for the development of all lymphomas after the diagnosis of chronic pruritus compared to controls.

Presented is the lymphoma risk for all lymphoma, cutaneous T cell lymphoma (CTCL) and systemic lymphoma stratified by sex (female/male) and ancestry (Black, Hispanic, White) after the diagnosis of chronic pruritus (CP) compared to patients without chronic pruritus (controls) using the Kaplan–Meier method.

| Outcome | Strata | CP | | | Controls | | | Log-Rank p | HR | 95%-CI |
| --- | --- | --- | --- | --- | --- | --- | --- | --- | --- | --- |
|  |  | n= | Outc-omes | % | n= | Out-comes | % |  |  |  |
| All lymphoma | Female | 698,588 | 1,607 | 0.230 | 698,588 | 570 | 0.082 | <0.0001 | 2.848 | 2.588-3.133 |
|  | Male | 307,573 | 1,262 | 0.410 | 307,573 | 420 | 0.137 | <0.0001 | 3.132 | 2.805-3.498 |
| CTCL | Female | 698,588 | 194 | 0.028 | 698,588 | 29 | 0.004 | <0.0001 | 6.744 | 4.565-9.964 |
|  | Male | 307,573 | 183 | 0.059 | 307,573 | 23 | 0.007 | <0.0001 | 8.218 | 5.326-12.678 |
| Systemic lymphoma | Female | 698,588 | 1,454 | 0.208 | 698,588 | 551 | 0.079 | <0.0001 | 2.665 | 2.416-2.94 |
|  | Male | 307,573 | 1,113 | 0.362 | 307,573 | 404 | 0.131 | <0.0001 | 2.873 | 2.564-3.22 |
|  |  |  |  |  |  |  |  |  |  |  |
| All lymphoma | Black | 192,135 | 469 | 0.244 | 192,135 | 134 | 0.070 | <0.0001 | 3.479 | 2.871-4.215 |
|  | Hispanic | 99,402 | 182 | 0.183 | 99,402 | 56 | 0.056 | <0.0001 | 3.240 | 2.402-4.372 |
|  | White | 613,953 | 1,976 | 0.322 | 613,953 | 709 | 0.115 | <0.0001 | 2.947 | 2.705-3.211 |
| CTCL | Black | 192,135 | 82 | 0.043 | 192,135 | 10 | 0.005 | <0.0001 | 8.173 | 4.239-15.758 |
|  | Hispanic | 99,402 | 19 | 0.019 | 99,402 | 10 | 0.010 | 0.002 | 4.741 | 1.613-13.936 |
|  | White | 613,953 | 224 | 0.036 | 613,953 | 38 | 0.006 | <0.0001 | 6.205 | 4.4-8.752 |
| Systemic lymphoma | Black | 192,135 | 405 | 0.211 | 192,135 | 127 | 0.066 | <0.0001 | 3.168 | 2.596-3.867 |
|  | Hispanic | 99,402 | 162 | 0.163 | 99,402 | 52 | 0.052 | <0.0001 | 3.106 | 2.272-4.244 |
|  | White | 613,953 | 1,793 | 0.292 | 613,953 | 681 | 0.111 | <0.0001 | 2.784 | 2.549-3.041 |

**Table S2**. Sex- and ancestry-stratified risk for the development of all lymphomas after the diagnosis of chronic prurigo compared to controls.

| Outcome | Strata | CPG | | | Controls | | | Log-Rank p | HR | 95%-CI |
| --- | --- | --- | --- | --- | --- | --- | --- | --- | --- | --- |
|  |  | n= | Out-comes | % | n= | Out-comes | % |  |  |  |
| All lymphoma | Female | 73,244 | 194 | 0.265 | 73,626 | 74 | 0.101 | 0.000 | 2.711 | 2.074-3.544 |
|  | Male | 50,439 | 200 | 0.397 | 50,439 | 60 | 0.119 | 0.000 | 3.359 | 2.517-4.483 |
| CTCL | Female | 73,592 | 24 | 0.033 | 73,626 | 10 | 0.014 | 0.000 | 24.601 | 3.328-181.848 |
|  | Male | 50,439 | 34 | 0.067 | 50,439 | 10 | 0.020 | 0.000 | 17.082 | 4.104-71.101 |
| Systemic lymphoma | Female | 73,267 | 180 | 0.246 | 73,626 | 74 | 0.101 | 0.000 | 2.515 | 1.919-3.297 |
|  | Male | 50,439 | 173 | 0.343 | 50,439 | 58 | 0.115 | 0.000 | 3.006 | 2.233-4.047 |
|  |  |  |  |  |  |  |  |  |  |  |
| All lymphoma | Black | 17,305 | 71 | 0.410 | 17,382 | 19 | 0.109 | 0.000 | 3.689 | 2.223-6.12 |
|  | Hispanic | 8,373 | 20 | 0.239 | 8,409 | 10 | 0.119 | 0.003 | 3.949 | 1.482-10.523 |
|  | White | 82,335 | 280 | 0.340 | 82,938 | 102 | 0.123 | 0.000 | 2.830 | 2.256-3.55 |
| CTCL | Black | 17,372 | 16 | 0.092 | 17,382 | 10 | 0.058 | 0.000 | 15.883 | 2.106-119.764 |
|  | Hispanic | 8,405 | 10 | 0.119 | 8,409 | 0 | 0.000 | 0.160 |  |  |
|  | White | 82,886 | 36 | 0.043 | 82,938 | 10 | 0.012 | 0.000 | 7.348 | 2.883-18.725 |
| Systemic lymphoma | Black | 17,311 | 58 | 0.335 | 17,382 | 18 | 0.104 | 0.000 | 3.173 | 1.87-5.384 |
|  | Hispanic | 8,376 | 18 | 0.215 | 8,409 | 10 | 0.119 | 0.007 | 3.551 | 1.318-9.564 |
|  | White | 82,370 | 257 | 0.312 | 82,938 | 98 | 0.118 | 0.000 | 2.702 | 2.141-3.411 |

Presented is the lymphoma risk for all lymphoma, cutaneous T cell lymphoma (CTCL) and systemic lymphoma stratified by sex (female/male) and ancestry (Black, Hispanic, White) after the diagnosis of chronic prurigo (CPG) compared to patients without chronic prurigo (controls) using the Kaplan–Meier method.

**Figure S1**. Risk for the lymphoma development after the diagnosis of chronic pruritus compared to controls.

Presentation of the risk for the development of all lymphoma (A), cutaneous T cell lymphoma (B) or systemic lymphoma (C) in patients with chronic pruritus (CP, blue line) over a period of 5 years compared to patients without a diagnosis of CP (controls, red line) as assessed by the Kaplan-Meier method (lines show mean survival with 95%-confidence intervals).

**Figure S2**. Risk for the lymphoma development after the diagnosis of chronic prurigo compared to controls.

Presentation of the risk for the development of all lymphoma (A), cutaneous T cell lymphoma (B) or systemic lymphoma (C) in patients with chronic prurigo (CPG, blue line) over a period of 5 years compared to patients without a diagnosis of CPG (controls, red line) as assessed by the Kaplan-Meier method (lines show mean survival with 95%-confidence intervals).

**Figure S3.** Risk of systemic lymphoma in chronic pruritus patients compared to controls.

The figure presents the hazard ratio (HR) of lymphoma development (all lymphoma, cutaneous T cell lymphoma (CTCL) and systemic lymphoma) after the diagnosis of chronic pruritus (cases) compared to controls (patients without chronic pruritus) as examined in the primary analysis (5 years follow-up) as well as with 1- and 10-years follow-up using the Kaplan-Meier method.

**Figure S4.** Risk of systemic lymphoma in chronic prurigo patients compared to controls.

The figure presents the hazard ratio (HR) of lymphoma development (all lymphoma, cutaneous T cell lymphoma (CTCL) and systemic lymphoma) after the diagnosis of chronic prurigo (cases) compared to controls (patients without chronic prurigo) as examined in the primary analysis (5 years follow-up) as well as with 1- and 10-years follow-up using the Kaplan-Meier method.
